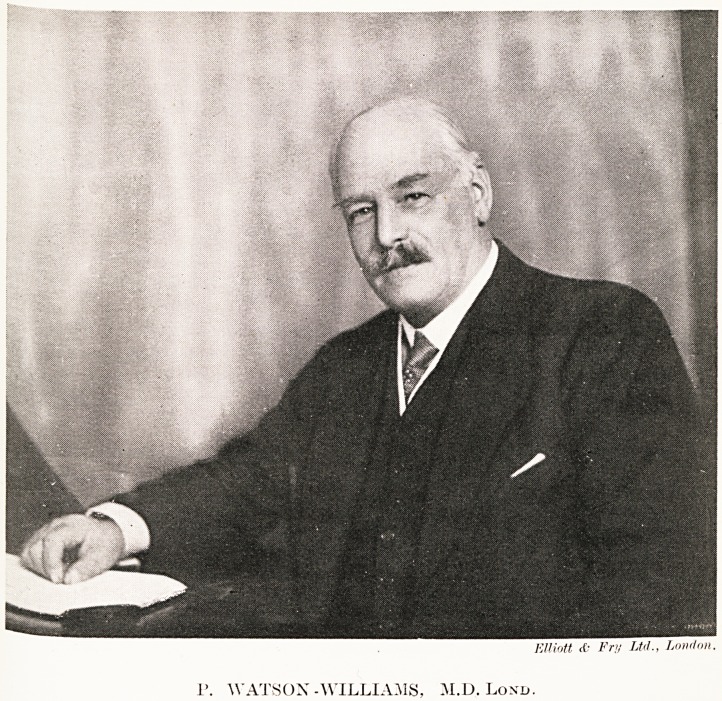# Patrick Watson-Williams

**Published:** 1938

**Authors:** 


### Obituary

### PATRICK WATSON-WILLIAMS.

DR. PATRICK WATSON-WILLIAMS died at his home in Rodney Place, Clifton, on 14th November, at the age of 77. *The Lancet* in a memoir of him said: "By his death we have lost a most valuable and enterprising member of that small band of pioneers who in this country changed oto-rhino-laryngology from a casual occupation to a highly developed speciality." Patrick Watson-Williams was born in Clifton, where his father, Dr. Eubulus Williams had practised for many years. He was educated at Clifton College and University College, Bristol, qualifying as M.R.C.S. in 1884, and taking the M.B. (London) with honours in 1885. He worked as a student at the Bristol

*Elliott & Fry Ltd., London.*

P. WATSON-WILLIAMS, M.D. LOND.

Royal Infirmary and remained on the staff there during the remainder of his life. He held the offices of house physician in 1887, assistant physician in 1888, and physician in 1905. During his long term as assistant physician he turned his attention to rhino-laryngology and acted as clinical assistant at the London Throat and Ear Hospital and the Central London Hospital.

He wrote (with Sir Felix Semon) the chapters on diseases of the throat in Allbutt's *System of Medicine* and was responsible for the sections in the *Medical Annual* on these diseases from 1890 onwards. In 1892 he published his *Diseases of the Upper Respiratory Tract,* which met with immediate success and carried his reputation far beyond the limits of his own country.

From 1897 to 1905 he was physician to Clifton College. In 1906 a new department for diseases of the throat and nose was established at the Bristol Royal Infirmary, of which he was placed in charge. He worked as head of this department until 1921, when he retired from the active staff and was elected consulting surgeon to the department, which after 1910 had been enlarged to include diseases of the ear. He had helped his friend and colleague, Sir Felix Semon, to found the Laryngological Society of London, which later became the Laryngological Section of the Royal Society of Medicine, and of this section he was president in 1910. In 1911 he represented the Society at the Berlin International Laryngological Congress. He was also president of the Laryngological Section of the British Medical Association in 1906. In 1925 he delivered the Semon lecture at London University on "The Relation of Chronic Nasal Sinus Infection to Septic Psychoses." In 1927 he was *Président d'Honneur* of the *Societé française d'Otologie et Laryngologie* in Paris. In 1934 the University of Bristol (whose degree of M.D. he had taken *ad eundem gradum* in 1912) recognized his distinguished services to his university and medicine in general by bestowing on him the M.D. degree *honoris causa.*

During the Great War Watson-Williams was consultant for diseases of the ear, nose and throat to the military hospitals in the Southern Command, with the rank of major.

The Bristol Medico-Chirurgical Society was deeply indebted to him. Not only was he in constant attendance at their meetings and a frequent contributor to discussions, but from 1900 to 1912 he was assistant editor and from 1912 to 1926 he was editor of this *Journal.* During the difficult days of the war and the period immediately following, his indomitable energy kept the *Journal* alive. Just before his retirement from the editorial chair in 1926 he succeeded in changing the *format* of the *Journal*, announcing the change in a characteristic editorial note : " With this issue (Spring, 1926) the Society's *Journal* appears in larger pages and type, which it is believed will enhance its value to our readers, in that the matter is presented in a better form, perhaps more worthy of the intimate association of the Medico-Chirurgical Society with the University of Bristol . . . With the completion of this issue the Editor resigns his charge with gratitude to the Committee for appointing him to this office for so many years past." The improvements which he then introduced were largely due to his knowledge of the technical aspects of printing and illustration. On 13th October, 1926, he was elected an Honorary Member of the Society.

" A. J. W.," writing of him in the *British Medical Journal*, summed up Dr. Watson-Williams's life into three periods :—

The initial phase was that of the physician taking an interest in laryngology, and particularly in helping it to evolve into a recognized specialized branch of surgery. In the second phase, having appreciated the wide possibilities open to the laryngologist by the evolution of modern surgery, he threw himself with extraordinary perseverance, ingenuity, and intelligence into the elaboration of the technique of operations, particularly those designed for the treatment of infections of the nasal accessory sinuses. As a result of this phase of his work instruments and methods devised by him are in use in all parts of the world. The third phase is represented by a combination of the outlook of the general physician and the operating surgeon. Here he set himself to emphasize the neglected close association between infection, particularly in the region of the nose and throat, and general constitutional disease, his opinions on this subject being well in advance of his time. He has left behind him many contributions to medical literature, and, apart from articles in the various journals, he wrote a textbook, *Diseases of the Upper Respiratory Tract*, and articles on diseases of the nose, throat, and trachea in Clifford Allbutt's *System of Medicine*. More recently, as evidence of the third phase, he wrote a book, *Chronic Nasal Sinusitis and its Relation to General Medicine*. As an artist he was highly accomplished, evidence of this gift being furnished by the many illustrations in his work which emanated from his own pencil. It was a real pleasure to watch him rapidly sketch pathological conditions which he might be demonstrating.

His old friend and colleague, Dr. James Swain, writes :—

Watson-Williams' career seems to be an illustration of the ancient proverb that "Chance contrives better than we ourselves," for when he was a resident officer at the Bristol Royal Infirmary he was an eligible candidate for the newly-formed post of Honorary Obstetric Physician.

In those days appointments to the medical staff were obtained by the votes of the Trustees, and there is little doubt that Watson-Williams would have been chosen had it not been for the questionable methods adopted by some of those who were unfavourable to his candidature, in consequence of which he withdrew his application.

This was a severe blow at the beginning of his professional work, but it proved to be a blessing in disguise, for, with characteristic pluck and determination, he decided to devote his energies to another branch of medicine, and the foundation of his life's work was inaugurated when he was appointed Assistant Physician to the Bristol Royal Infirmary and in charge of the Throat Department.

He was, however, a man of long views and saw the desirability of establishing a special department for Diseases of the Ear, Nose and Throat. His advocacy was ultimately rewarded by the formation of this department, of which he was elected to take charge, and in this position he worked with untiring devotion to his speciality and gained an international reputation.

Many of us have to be content with the pursuit of our ideals without attaining them, but Watson-Williams achieved his object and deserved to do so.

> " 'Tis not in mortals to command success,
> But we'll do more, Sempronius, we'll deserve it."

He sought relaxation in the casting of a fly or in the handling of a billiard cue and was a good exponent of both. He also had a natural ability for drawing and could etch his own Christmas cards and trace the predominant expression in a portrait with a few strokes of his pencil. He had a generous disposition, was "given to hospitality," and fully enjoyed his position as host at the scrumptious banquets which he gave on various public occasions. In private life, those of us who were privileged to sit at his table know with what courtesy he presided and how solicitous he was that his guests should receive freely of all that he could provide. This is not the place to refer to his copious contributions to medical literature, but the writer is glad to remember that he had the pleasure of revising the proof sheets of the first edition of Watson-Williams' book on *Diseases of the Upper Respiratory Tract*, published in in 1894. His many friends will miss his kindly welcome and genial smile: he leaves behind an honoured name in the annals of Bristol and its medical school.

In 1889 Dr. Watson Williams married Margaret, a daughter of the late Dr. Edward Long Fox, of Clifton. He leaves a widow, three daughters and two sons, one of whom is in charge of the department at the Royal Infirmary which his father established.

## Figures and Tables

**Figure f1:**